# Clinical Implication and the Hereditary Factors of NM23 in Hepatocellular Carcinoma Based on Bioinformatics Analysis and Genome–Wide Association Study

**DOI:** 10.1155/2018/6594169

**Published:** 2018-12-18

**Authors:** Chengkun Yang, Chuangye Han, Xiangkun Wang, Xiwen Liao, Xiaoguang Liu, Wei Qin, Long Yu, Guangzhi Zhu, Hao Su, Sicong Lu, Zhiwei Chen, Tingdong Yu, Zhen Liu, Ketuan Huang, Zhengtao Liu, Yu Liang, Jianlu Huang, Tao Peng

**Affiliations:** ^1^Department of Hepatobiliary Surgery, The First Affiliated Hospital of Guangxi Medical University, Nanning, 530021, Guangxi Province, China; ^2^Department of Hepatobiliary Surgery, Affiliated Hospital of Guangdong Medical University, Zhanjiang, 524001, Guangdong Province, China; ^3^Hepatobiliary and Pancreatic Surgery, The First Affiliated Hospital of Zhengzhou University, Zhengzhou, 450000, Henan Province, China; ^4^Department of Hepatobiliary Surgery, Third Affiliated Hospital of Guangxi Medical University, Nanning 530031, Guangxi Province, China

## Abstract

NM23 expression is closely associated with hepatocellular carcinoma (HCC) recurrence, but the hereditary factors influencing NM23 levels are unknown. Using public database, the diagnostic value of* NM23* in HCC was investigated. A total of 424 hepatitis B virus- (HBV-) related HCC patients were enrolled to perform a genome–wide association study for identifying candidate variants associated with NM23 expression level. Additionally, a logistic regression model, haplotypes, and survival analysis were performed in the subsequent analysis. We identified high NM23 expression levels that have a diagnostic accuracy in HCC tissues and had a poor recurrence-free survival in HBV-related HCC patients. Variants near Psoriasis susceptibility 1 candidate 1 (*PSORS1C1*) and StAR related lipid transdomain containing 3 (*STARD3*) are associated with NM23 expression. The* PSORS1C1* haplotype TGCACA and the* STARD3* haplotype GG have favorable cumulative effects on NM23 expression. Further, variants in PSORS1C1 were associated with either overall survival (rs556285588, rs3095301, and rs3131003) only or overall survival and recurrence-free survival (rs560052000 and rs541820233) both in HCC patients. Our findings suggested that variants at the* PSORS1C1* and* STARD3* loci play an important role in* NM23* regulation. Moreover, variants in* PSORS1C1* are potential biomarkers for the prediction of postoperative clinical outcomes in HBV-related HCC patients. Thus, variants in* PSORS1C1* and* STARD3* are associated with NM23 expression and clinical outcomes of HBV-related HCC patients, which may be regarded as potential biomarkers for this disease.

## 1. Introduction

Primary liver cancer (PLC) is a common malignant neoplasm, with an estimated 854 000 incident cases and 810 000 deaths globally in 2015, contributing to 20 578 000 disability–adjusted life-years [1]. Hepatocellular carcinoma (HCC) comprises 85%–90% of PLC, and the 5-year survival after resection for early–stage HCC ranges from 17 to 53% with recurrence rate as high as 70% [2, 3]. HCC is a multifactorial disease involving a complex interplay between genetic and environmental factors. Epidemiological studies indicated that the major etiological factors affecting HCC include hepatitis B virus (HBV), hepatitis C virus (HCV), aflatoxin exposure, excessive alcohol intake, liver flukes, and cirrhosis [4, 5]. The onset and development of HCC are generally considered to be the consequence of a multistepped process involving the activation of oncogenes and inactivation of tumor suppressor genes.

NME/NM23 nucleoside diphosphate kinase 1 (*NM23*), also known as* NME1*, is first reported as an antitumor–metastasis gene that was correlated with metastasis in murine melanoma [6]. NM23 protein expression was associated with cell–cell adhesion, cell migration, proliferation, and invasion depth [7, 8]. Several studies suggested that NM23 expression was inversely proportional to the aggressive metastatic behavior of melanoma as well as gastric, colon, and breast carcinomas [9]. Metastasis is the major cause of morbidity and mortality in individuals with HCC. It has also been reported that the expression of NM23 in tumor tissues is correlated with the occurrence of metastasis and length of survival of HCC patients [10]. Moreover, some studies reported that NM23 expression was upregulated in HCC neoplastic tissue as compared to nontumor tissue [11, 12]. Wei–lu et al. [13] showed that transcatheter arterial chemoembolization (TACE) enhanced the expression of NM23 in HCC patients. However, little is known about the genetic determinants of NM23 expression in HCC.

Recently, genome–wide association studies (GWAS) have become an efficient method to study the molecular genetics of HCC development and progression [14]. In the present study, we investigated the diagnostic value of* NM23* in HCC and performed a GWAS to explore the association between genetic variants and NM23 expression in HBV-related HCC, aiming to identify a novel therapeutic target for* NM23* regulation. The* NM23* isoforms (NM23-H1 or NM23-H2, also called* NME1* or* NME2*) are heterogeneous in the process of metastasis of HCC; however,* NME1* is recommended as one of immunohistochemical markers associated with biological properties of HCC in China. Thus, our study focuses on the expression of this specific isoform.

## 2. Materials and Methods

### 2.1. Evaluation of Diagnostic Value for NM23 Expression in HCC

The NM23 specific isoform expression in HCC was obtained from GEO (http://www.ncbi.nlm.nih.gov/geo/) and Oncomine (https://www.oncomine.com/). The criteria used to determine study eligibility were as follows: (1) human species; (2) histopathology confirmed with HCC; (3) availability NME1 expression in HCC and paracancerous; (4) use of prospective or retrospective cohort design with a clearly defined source population and justify all excluded eligible cases; and (5) selection of the latest and most complete study to avoid duplication. NM23 expression in tumor and nontumor tissues was presented as mean and standard deviation (SD) and compared by Student's test. The receiver operating characteristic (ROC) curve was performed to identify the diagnostic value of* NM23* in patients with HCC. The area under the curve (AUC) value was calculated for evaluating the predictive accuracy and discriminative ability of ROC.

### 2.2. Study Population

A total of 424 patients were enrolled at the First Affiliated Hospital of Guangxi Medical University (Guangxi, China) from 2005 to 2013. All HBV-related HCC subjects were histopathologically confirmed after hepatectomy. This study was approved by the Ethics Committee of the First Affiliated Hospital of Guangxi Medical University.

### 2.3. Immunohistochemistry

All tumor tissues were immunohistochemically stained for NME1 by full–time pathologists according to routine processes and the guidelines for standardized pathological diagnosis of primary liver cancer. Supersensitive reagents and mouse anti-human NME1 monoclonal antibody (clone OTI4G3) and streptavidin-peroxidase anti-human kit were purchased from OriGene (Beijing OriGene Technologies, Inc., China). The immunohistochemical staining was carried out by following the manufacturer's instructions of the kit. For the negative control, the primary antibody was replaced by phosphatebuffered saline/Tween. Positive staining for the NM23 protein appeared as yellow–brown particles.

**Figure 1:**
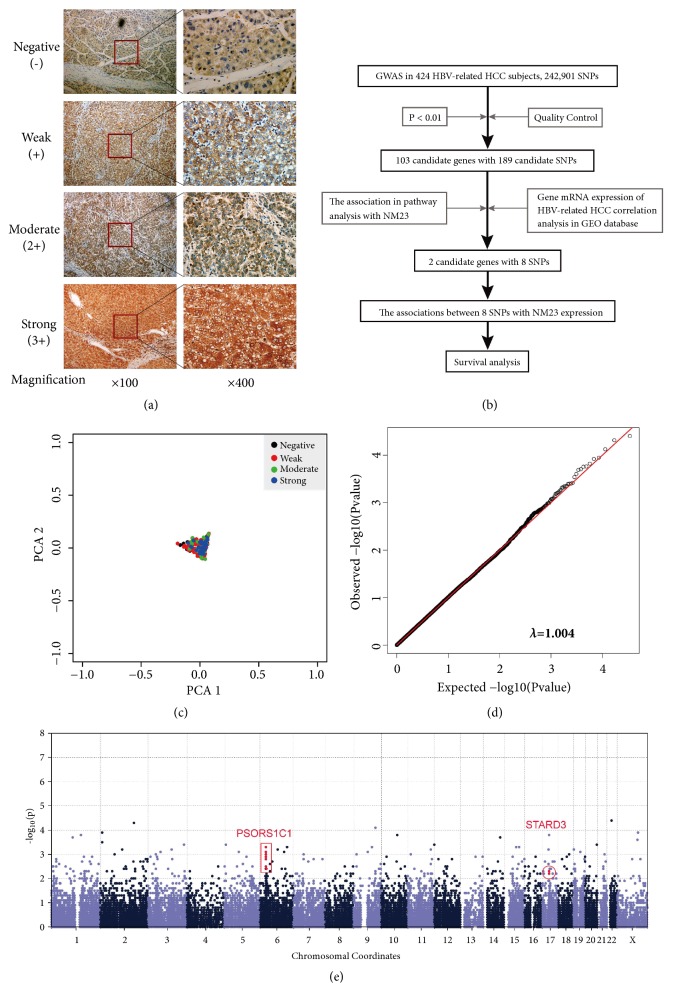
(a) Representative staining of NM23 protein (negative, weak, moderate, and strong) in HCC tissues. Original magnifications were ×100 and ×400, respectively. (b) The flowchart depicts the process of the GWAS, screening the candidate SNPs, and association analysis in this study. (c) Principal components analysis (PCA) for ancestry and population stratification implemented in the EIGENSOFT package. The blue dots represent negative NM23 expression, the red dots represent weak NM23 expression, the green dots represent moderate NM23 expression, and the blue dots represent strong NM23 expression. (d) Quantile–Quantile (Q–Q) plot for the GWAS results. The genomic inflation factor was calculated by MATLAB 7.0 based on the* P–*value, and the genomic control inflation factor (*λ*) is 1.004. (e) The Manhattan plot for the association analysis.

The criteria used to analyze NM23 expression were based on the number and staining intensity of the stained cells [16, 17]. Briefly, a mean percentage of positive tumor cells was determined in at least five areas at ×400 magnification (50–250 cancer cells per area) and assigned scores as follows: 0: ≤5%; 1: 6%–25%; 2: 26%–50%; 3: 51%–75%; and 4: ≥76%. For convenience of assessment, the intensity of immunostaining was scored following a quantitative principle of proportion: 0, negative, equal to the negative control; 1, weak, cytoplasmic stain slightly darker than the negative control; 2, moderate, defined as an intensity between 1 and 3; 3, strong staining, darker than the positive control. Each sample was processed along with a negative and positive control tissue as references. The sum of the staining intensity and staining extent scores (0–7) was used as the final staining score; that is, a final staining score of 0–1, 2–3, 4–5, or 6–7 was considered to be negative (–), weak (+), moderate (++), or strong (+++) expression, respectively (the representative staining of NM23 in HCC tissues is shown in Figure 1(a)). The results were examined independently and checked collectively by two pathologists who were blinded to the clinicopathological variables. Consequently, the total number of negative, weak, moderate, and strong NM23 expression samples was 43, 285, 48, and 48, respectively.

### 2.4. DNA Extraction and Genotyping

DNA was extracted from HCC specimens (including paracancer tissues) using the TIANamp Genomic DNA Kit (Tiangen Biotech, Beijing, China) according to the manufacturer's instructions. DNA yield and purity were measured by the NanoDrop2000 system (Thermo Fisher Scientific, Waktham, MA, USA).

We genotyped all samples on the Illumina Human Exome BeadChip-12-1_A, which includes 242,901 markers focused on protein-altering variants. Genotype calling was carried out using Genotyping Module v1.0 in GenomeStudio. A total of 50 samples (over 10%) were randomly selected using random number table and sequenced for candidate loci by ABI Prism 3100 (Applied Biosystems, Shanghai Sangon Biological Engineering Technology & Services, Shanghai, China), yielding a 100% concordance rate with the genotyped variants (the SNP Sequenced primers were showed in Supplementary Table S1).

### 2.5. Patients Follow-Up

All patients were followed up after discharge until death or the last time of follow-up (September 2014) by personal or family contacts. The median follow-up time for the 424 patients was 42 months (ranging from 4 to 125 months), and the median survival time (MST) was 30 months. Overall survival (OS) and recurrence-free survival (RFS) were calculated.

### 2.6. Statistical Analysis

#### 2.6.1. Study Design and Analysis

We performed a GWAS following the process displayed in the flowchart (Figure 1(b)). A large set of single nucleotide polymorphisms (SNPs) was identified using the GWAS array. Then, candidate SNPs were selected using association and pathway analyses. Finally, for these candidate SNPs, the association with the clinical outcomes of HCC patients was evaluated by survival analysis in SPSS.

#### 2.6.2. Quality Control (QC)

Population stratification was estimated by a principal components analysis (PCA), implemented by the EPACTS package in MATLAB 7.0 (MathWorks, Natick, MA, USA). A quantile–quantile (Q–Q) plot was used to evaluate the potential impact of population stratification. QC filtering was performed with PLINK version 1.07, R v. 3.0.1 (https://www.r-project.org/) and the EIGENSOFT package (http://genetics.med.harvard.edu/reich/Reich_Lab/Software.html) as previously described [18, 19]. Finally, candidate SNPs with acceptable quality were analyzed in a discovery GWAS for NM23 expression.

### 2.7. GWAS

#### 2.7.1. Association Analysis

A linear regression model was used to test the association of quantitative trait (intensity of NM23 expression) with the SNPs that passed QC using the EPACTS package version 3.2.6 [20], adjusting for age, gender, race, body mass index (BMI), smoking status, drinking status, preoperative TACE, Barcelona Clinic Liver Cancer (BCLC) stage, liver cirrhosis, and Edmondson classification as covariates, and an additive model for allelic effect was assumed. The* p*–value threshold for the GWAS was 0.01. Baseline variables in intensity of NM23 expression were evaluated using the Chi–square test or Fisher's exact test. Afterward, we performed ordinal logistic regression analysis to assess the cumulative effect of baseline variables and candidate SNP genotypes on NM23 expression by computing odds ratios (ORs) and 95% confidence intervals (CIs). The reliability of all results was assessed by the test of parallel lines. The correlation of mRNA expression of candidate genes and* NM23* was measured by correlation analysis. Differences of mRNA expression of genes between HCC and adjacent normal tissues were verified by Student *t* test.

#### 2.7.2. Bioinformatics Analysis

We selected all genes containing candidate SNPs to search for gene–gene interactions. The signaling pathway network diagram was performed with GeneMANIA Software [21]. Moreover, local linkage disequilibrium (LD) and recombination patterns nearby candidate SNPs were analyzed using LocusZoom [22] to create regional association plots. The LD values between SNPs were analyzed by the Haploview 4.2 program. The SNPs expression Quantitative Trait Locus (eQTL) mapping was analyzed in GTEx portal (https://www.gtexportal.org).

### 2.8. Survival Analysis

The Kaplan–Meier lifetable method was used to calculate OS and RFS rates, and differences in survival rates were estimated using a generalized log-rank test. The association between candidate gene and clinical outcomes of HCC patients was analyzed using Gene Expression Profiling Interactive Analysis (GEPIA) (http://gepia.cancer-pku.cn/) [23] based on the Cancer Genome Atlas (TCGA) database. A Cox proportional hazard regression model was used to calculate hazard ratios (HR) and 95% CIs. All statistical analyses were two–sided and performed using SPSS version 24.0 (SPSS, Chicago, IL, USA). A* P*–value of less than 0.05 was considered statistically significant. The survival curves were depicted by GraphPad 7.01 (GraphPadSoftware, Inc., Sandiego, CA, USA).

## 3. Results

### 3.1. Public Database Analysis

**Figure 2:**
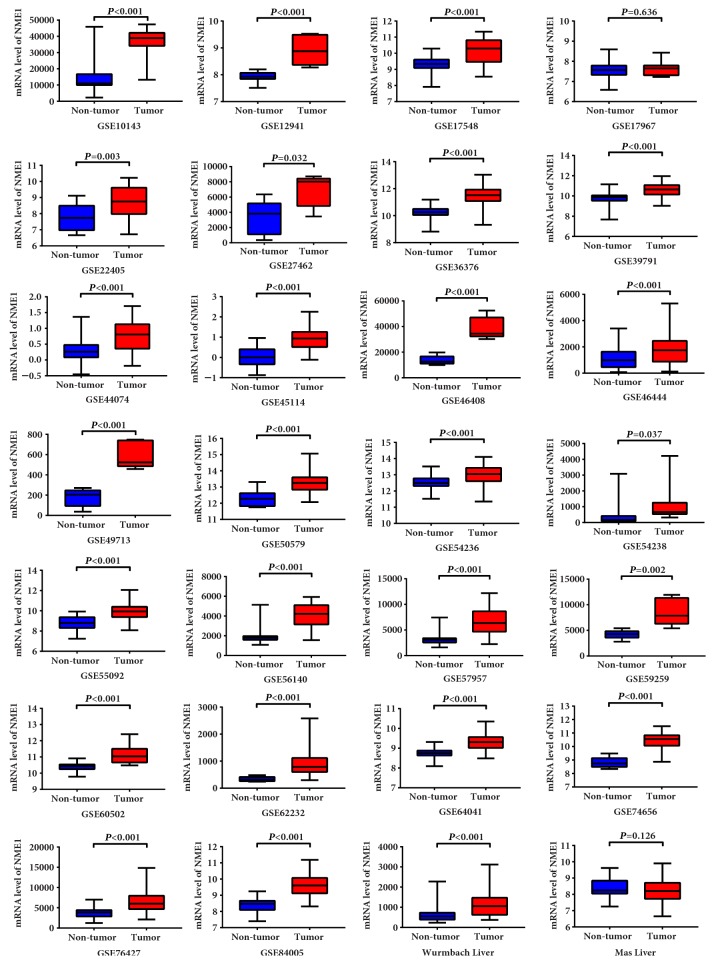
The NM23 expression of HCC and nontumor tissues in public datasets.

**Figure 3:**
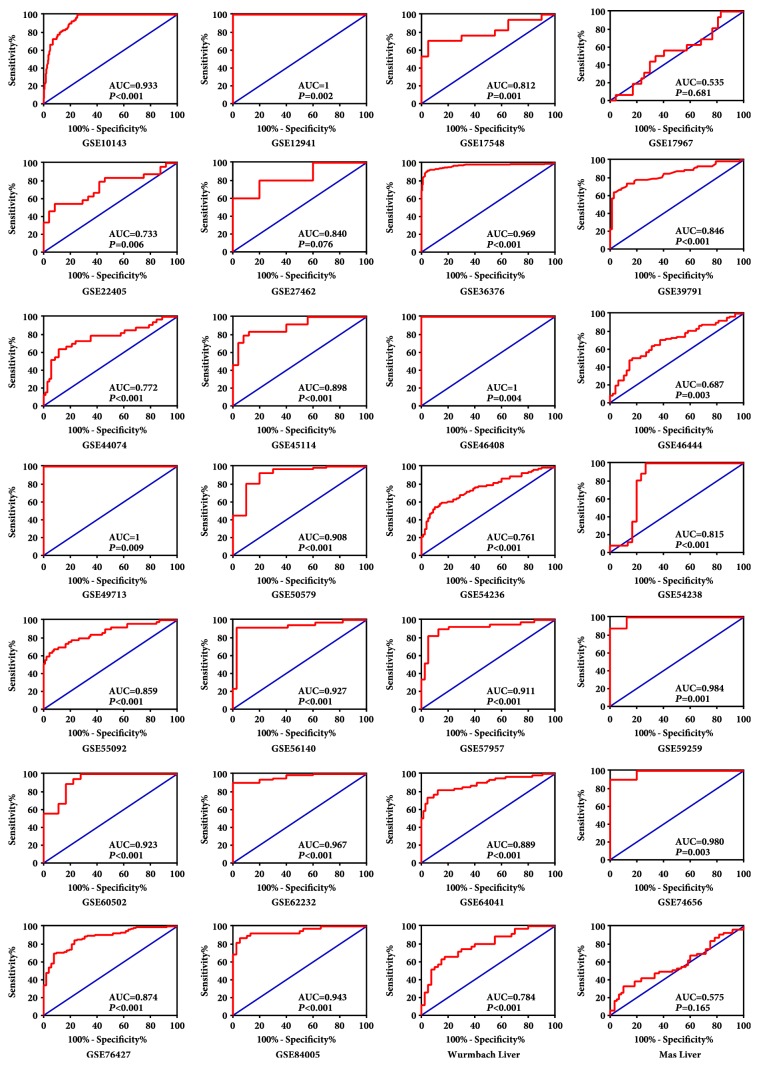
The ROC analysis of NM23 expression in public HCC and nontumor tissues datasets. Blue: identity cure; red: sensitivity cure for the differentiation of HCC from nontumor tissues.

A total of 28 datasets were enrolled in this study from GEO and Oncomine (Supplementary Table S2). NM23 expression was elevated in HCC tissues as compared to non–tumor tissues in 26 of 28 datasets (Figure 2). The ROC analysis of NM23 expression in HCC datasets indicates high NM23 mRNA level had high accuracy in distinguishing tumor from non–tumor tissues (the AUC of the ROC curves in most of datasets were >0.70, Figure 3).

### 3.2. GWAS

#### 3.2.1. Baseline Characteristics

The groups were similar with respect to most of the characteristics (*P*>0.05, Table 1). Age, BMI, regional invasion, and antiviral therapies were significantly different (*P*<0.05, Table 1) based on the results of the Chi–square test or Fisher's exact test. The univariate ordinal regression results show BMI≤25 (OR=1.75, 95% CI=1.05–2.93, and Table 1) might be a favorable factor for positive expression of NM23, but ethnic Han ancestry (OR=0.62, 95% CI=0.41–0.95), serum alpha–fetoprotein (AFP)≤400 ng/ml (OR=0.61, 95% CI=0.40–0.93), absence of regional invasion (OR=0.40, 95% CI=0.23–0.67), and antiviral therapies (OR=0.59, 95% CI=0.39–0.90) may associate with lower NM23 expression.

#### 3.2.2. QC

A total of 408 HBV-related HCC patients and 21,529 SNPs were included in further analysis after QC filtering. A PCA plot demonstrated that there were no outliers in this study population (Figure 1(c)). The genomic inflation factor (*λ*) in this study was 1.004 (Figure 1(d)).

#### 3.2.3. Association Analysis

Based on the results of the GWAS displayed in a Manhattan plot (Figure 1(e)), as well as mRNA expression and LD analysis, we identified that SNPs in/near the candidate genes “Psoriasis susceptibility 1 candidate 1” (*PSORS1C1*) and “StAR related lipid trans domain containing 3” (*STARD3*) were associated with the expression of NM23 in HBV-related HCC (Supplementary Table S3). The association analysis between these eight SNPs and NM23 expression showed that rs560052000, rs541820233, rs556285588, rs3131003, rs3095301, and rs3095302 were strongly associated with a cumulative effect on NM23 expression (Supplementary Table S4). Moreover, rs11869286–CC was associated with high NM23 expression, whereas rs1877031–GG was associated with lower NM23 expression and rs1877031–AG was associated with it when adjusted than rs1877031–AA.

#### 3.2.4. Pathway Analysis and Correlation Analysis in mRNA

**Figure 4:**
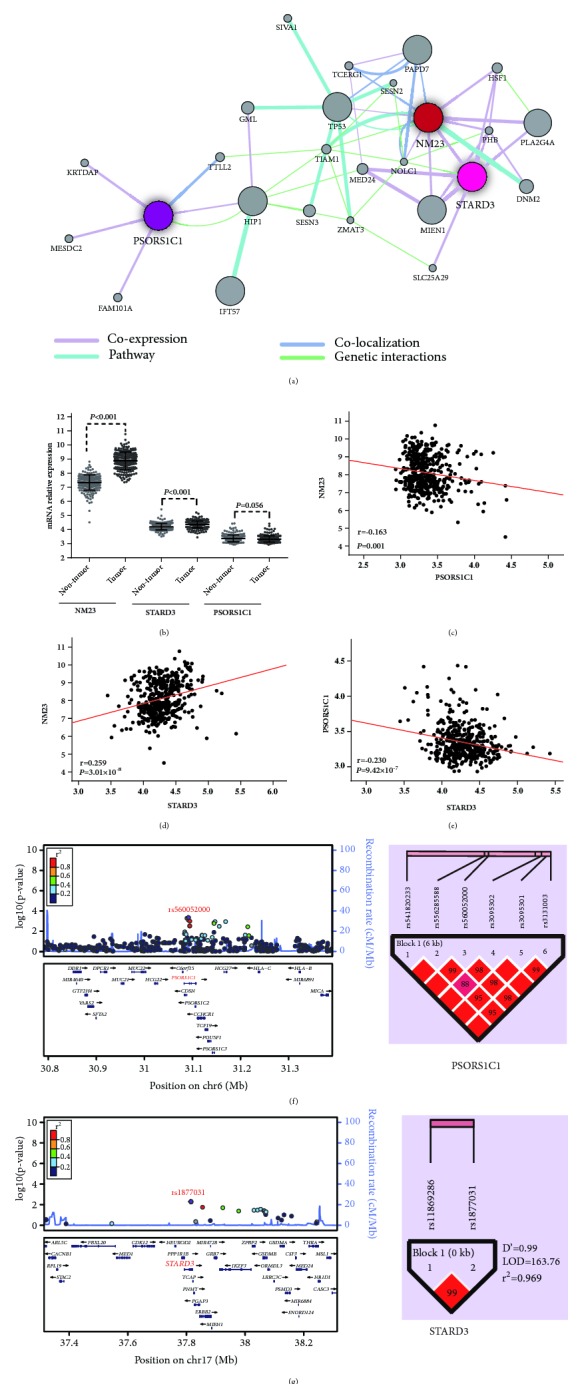
The association of* NM23*,* STARD3* and* PSORS1C1* in HCC patients. (a) Pathway network analysis for* NM23*,* STARD3*, and* PSORS1C1* in GeneMANIA. (b) A scatter plot for the mRNA expression of* NM23*,* STARD3*, and* PSORS1C1* between HCC and adjacent normal tissues.* NM23* and* STARD3* have a* P*<0.001, but* PSORS1C1* has a* P*=0.056. (c) Correlation analysis for the mRNA expression between* NM23* and* PSORS1C1*, r=-0.163,* P=*0.001. (d) Correlation analysis for the mRNA expression between* NM23* and* STARD3*, r=0.259, and* P=*3.01 ×10^−8^. (e) Correlation analysis for the mRNA expression between* PSORS1C1* and* STARD3*, r=-0.230, and* P*=9.42 ×10^−7^. All data are from the Gene Expression Omnibus (GEO accession: GSE14520). (f and g) LocusZoom plot for the analysis of local linkage disequilibrium (LD) and recombination patterns nearby* PSORS1C1* (f) and* STARD3* (g), ranging around 0.5 to 0.8 Mb. The vertical axis shows association* P*–values on the −log10 scale, and the right vertical axis shows the recombination rate. The horizontal axis shows the chromosomal position. The bottom of the plot shows the nearest gene. LD (r^2^) and recombination rates are estimated from the 1000 Genomes Project ASN data (March2012, build GRCh37/hg19). Haploview LD plot of* PSORS1C1* SNPs (n=6) (f) and* STARD3* SNPs (n=2) (g). Blocks represent SNPs in high LD. Greater color intensity and value inside each cell corresponds to a higher level of LD given by D'.

The signaling pathway network showed that* NM23* may interact with* STARD3* (Figure 4(a)). Additionally,* PSORS1C1* may interact with* NM23* through T–cell lymphoma invasion and metastasis 1 (*TIAM1*) and* TP53*. Further, we used data from the Gene Expression Omnibus (GEO accession: GSE14520) to analyze the mRNA expression of* PSORS1C1*,* STARD3*, and* NM23* between HCC and adjacent normal tissues. Downregulation of* PSORS1C1* was observed in the tumor tissues while* STARD3* and* NM23* gene expression increased, when compared with the adjacent normal tissues (Figure 4(b)). Correlation analysis was performed to account for the relationship among* PSORS1C1*,* STARD3*, and* NM23* expression. There was a statistically significant negative correlation between* PSORS1C1* and* NM23 *expression (r=-0.163,* P*=0.001, Figure 4(c)), but* STARD3* and* NM23* were positively correlated (r=0.259,* P*=3.01 × 10^−8^, Figure 4(d)).* PSORS1C1* expression was negatively related to* STARD3* expression (r=0.230,* P*=9.42 × 10^−7^, Figure 4(e)).

#### 3.2.5. LD and Haplotype Analysis

Haplotype analysis revealed that six SNPs of* PSORS1C1* and the two SNPs in* STARD3* showed a strong LD block in Haploview (Figures 4(f) and 4(g)). The regional association plots (Figures 4(f) and 4(g)) show the negative log* P*–values and LD patterns of these SNPs in the combined analysis. The* PSORS1C1* haplotypes CAGGTG and CAGACA and the* STARD3* haplotypes (CA+GA+CG) had a lower cumulative effect on the expression of NM23 (OR=0.44, 95% CI=0.30–0.65; OR=0.56, 95%=0.32–0.99; OR=0.65, 95% CI=0.47–0.91, respectively) (Table 2), compared to the* PSORS1C1* haplotype TGCACA and the* STARD3* haplotype GG, respectively.

#### 3.2.6. eQTL Analysis

The eQTL mapping was performed in a total number of 153 samples in GTEx portal. Rs1877031 and rs11869286 presented a negative eQTL relationship in liver samples (both normalized effect size=-0.25,* P*=2.1 × 10^−7^) while rs3095302 and rs3131003 had a positive eQTL association in liver tissues (both normalized effect size=0.54,* P*=1.3 × 10^−6^, Supplementary Figure S1).

### 3.3. Survival Analysis

#### 3.3.1. Distribution of Patient Characteristics and Clinical Outcomes Analysis

MST was calculated using the Kaplan–Meier method with a log-rank test in the different subgroups of baseline variables (Table 1). There were statistical differences among the subgroups in Child–Pugh classification A, BCLC stage A, BCLC stage B, single tumor nodes, tumor size, absence of intrahepatic metastasis, PVTT, radical resection, and insufficient of antiviral therapies in OS of HCC patients. In the RFS, Child–Pugh classification A, BCLC stage A, absence of intrahepatic metastasis, and PVTT had a longer time until recurrence (Table 1).

#### 3.3.2. Association between Candidate Genes and NM23 and Their Complication in HCC Patients

**Figure 5:**
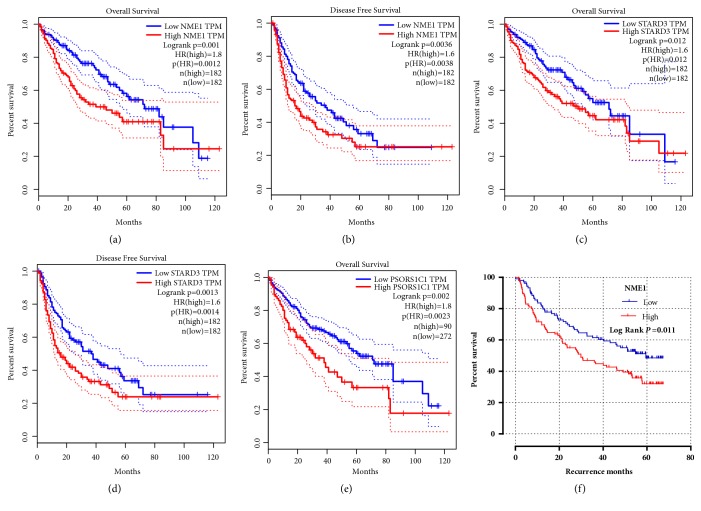
Kaplan–Meier survival analysis of* NM23*,* STARD3* and* PSORS1C1* in clinical outcomes of HCC patients. (a–d) The association between NM23 and STARD3 expression level and OS of HCC patients was analyzed in GEPIA. Cutoff value was set at median. (e) The association between NM23 expression level and RFS of HCC patients was analyzed in GEPIA. Cutoff value was set at quartile. (f) The association between NM23 expression level and RFS of HCC patients was analyzed in GSE14520. Cutoff value was set at median. Statistical differences were determined by log-rank test.

The prognostic value of* PSORS1C1*,* STARD3,* and* NM23* was evaluated in GEPIA and GSE14520. The cutoff value was set at median in STARD3 and NM23 and quartile in PSORS1C1. High STARD3 and NM23 expression level in patients with HCC had an unfavorable OS and RFS (Figure 5). Besides, high PSORS1C1 expression in HCC patients was a risk factor in OS (HR=1.8, P=0.00023; Figure 5). In HBV-related HCC patients of Guangdong cohort, high NM23 expression level was associated with RFS (Figure 5; HR=1.47, 95% CI=1.01-2.13, Supplementary Table S5).

#### 3.3.3. Association of PSORS1C1 and STARD3 SNPs with Clinical Outcomes in HBV-Related HCC Patients

**Figure 6:**
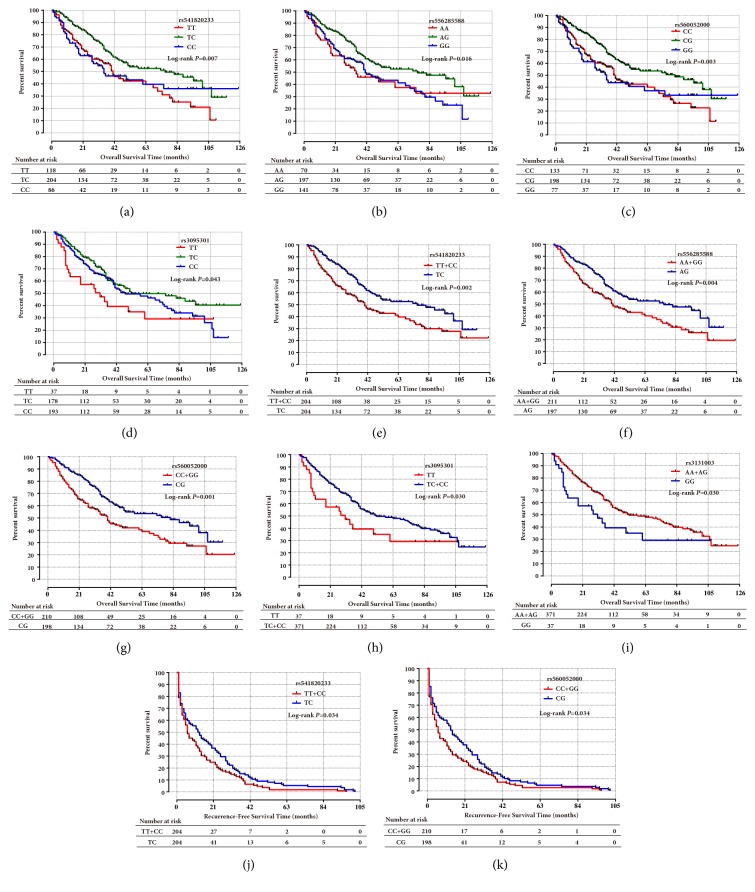
Kaplan–Meier survival analysis of SNPs in* PSORS1C1* (a–k) in clinical survival of HBV-related HCC patients after hepatic resection. There are statistical differences among the three groups (the genotypes of each SNP) (a–d) and two groups (the homozygote genotypes of each SNP were merged when comparing to the heterozygote genotype) (e–g, j–k). In particular, genotypes TC and CC at rs3095301 were merged (h), and genotypes AA and AG at rs3131003 were merged (i). Statistical differences were determined by log-rank test.

After hepatic resection, there was a significant difference in the MST of* PSORS1C1* SNPs rs541820233, rs556285588, rs560052000, and rs3095301 and when merged two of the genotypes in rs541820233–TT+CC, rs556285588–AA+GG, rs560052000–CC+GG, rs3095301–TC+CC, and rs3131003–AA+AG (Figures 6(a)–6(i), Supplementary Table S5). rs541820233 genotype TC (HR_OS_=0.64, 95% CI=0.47–0.87), rs556285588 genotype AG (HR_OS_=0.62, 95% CI=0.45–0.86), and rs560052000 genotype CG (HR_OS_=0.61, 95% CI=0.44–0.84) were associated with better survival. Additionally, when comparing the groups that were merged the homozygote genotypes, we found that people who harbored heterozygote genotype rs541820233–TC (HR_RFS_=0.69, 95% CI=0.50–0.96) and rs560052000 CG (HR_RFS_=0.67, 95% CI=0.48–0.93) may have a favorable prognosis in terms of RFS after hepatic resection (Figures 6(j)–6(k), Supplementary Table S6).

#### 3.3.4. Stratification Analysis of PSORS1C1 Associated with Clinical Outcomes

A Cox proportional hazard regression model was applied to perform stratified analysis and further assessed the relationship between the SNPs with OS. We found that* PSORS1C1* SNPs rs541820233–TC, rs556285588–AG, and rs560052000–CG were protective factors for most of the clinicopathological and oncological features analyzed (Supplementary Figure S2).

#### 3.3.5. Association of PSORS1C1 and STARD3 Haplotypes with Clinical Outcomes

The association between* PSORS1C1* and* STARD3* haplotypes and prognosis after hepatic resection was analyzed through a Cox proportional hazard regression model. However, there was no statistically difference between* PSORS1C1* haplotypes CAGGTG, CAGACA, and CGCACA+CGGGCA, when compared to TGCACA (Table 2). Similarly,* STARD3* haplotypes were not significantly different (CA+GA+CG; HR_OS_=0.97, 95% CI=0.76–1.24; HR_RFS_=1.18, 95% CI=0.74–1.88) versus the GG haplotype (Table 2).

## 4. Discussion

In this study, we investigated the diagnostic value of* NM23* and performed a GWAS to explore the association between hereditary factors with NM23 expression in HBV-related HCC patients in Guangxi. High NM23 expression showed a precise discrimination in HCC patients, with the AUC of the ROC curves in most of datasets more than 0.70. We detected variants in candidate genes* PSORS1C1* and* STARD3* fell into two strong LD blocks, with a cumulative effect on the expression intensity of NM23. Interestingly, we found that* PSORS1C1* variants associated with clinical outcomes in HBV-related HCC patients. In addition, multivariate Cox proportional hazard regression model analysis demonstrated that* PSORS1C1* SNPs rs541820233 and rs560052000 were associated with the OS and RFS of HCC patients, and heterozygous genotypes at these variants were associated with lower risk, implying that these SNPs may be independent prognostic indicators.

HCC is the third leading cause of cancer-related deaths worldwide [24]. Chronic HBV infection affects over 350 million people worldwide and remains one of the leading causes of cirrhosis, liver failure and HCC [25]. Different virus infections may cause variability and complexity of HCC, like HBV and HCV, resulting in different oncology characteristics. Some independent studies suggest that different viral proteins have critical roles in regulating NM23 functions and changing biological activities in cancer progression [26–28]. Run et al. [10] showed that the disease-free survival in HCC patients with negative NM23 expression was significantly poorer than that in patients with positive NM23 expression. By contrast, report by YB Liu et al. indicated the high NM23 expression in the group with high tendency to metastasis and recurrence and in patients with metastasis or recurrence during the follow-up [29]. In our study, high NM23 expression in HBV-related patients harbored an unfavorable RFS. However, some critical issues as well as host factors, including immunosuppression, somatic mutations, genetic predisposition, and exposure to carcinogens, have important contributory roles [30]. Our result suggested that variants of two immune disease-related genes were associated with NM23 expression in patients with HBV-related HCC.

*PSORS1C1* is located at 6p21.3, near the major histocompatibility complex (MHC) class I region. A regional association plot showed that* PSORS1C1* lies nearby human leukocyte antigen (HLA)–B and HLA–C. HLA is an important component of the MHC region. One study [31] reported that* PSORS1C1* was associated with HLA–independent systemic sclerosis. Additionally,* PSORS1C1* was found to be in strong linkage disequilibrium with the* HLA*–*DQB1* haplotype [31]. In our previous report, we identified that* HLA*–*DQB1* variants associated with OS in HCC patients [32]. The HLA gene family has also been associated with HBV-related HCC in recent studies [33, 34]. Our study showed that* PSORS1C1* variants are associated with NM23 expression. Further, we found that the rs560052000–GC, rs541820233–AG, rs556285588–TC, rs3131003–TT+TC, and rs3095301–AA+AG genotypes were associated with better OS in our study subjects. Stratified analysis demonstrated that rs560052000–GC, rs541820233–AG, and rs556285588–TC are protective genotypes in HBV-related HCC patients, having lower HRs for most clinicopathological factors. Although no associations were found in rs3095302 with survival outcomes, all six* PSORS1C1* SNPs were in strong linkage disequilibrium. The other haplotypes (CAGGTG, CAGACA, CGCACA, and CGGGCA) had a lower cumulative effect on the expression of NM23 when compared with the TGCACA haplotype. The LD of* PSORS1C1* with the HLA region may play a critical role. Tschiedel et al. [35] reported that* NM23* was identified as a novel HLA–A32 restricted tumor–associated antigen in chronic myeloid leukemia. Alternatively, the result of our pathway analysis showed that* PSORS1C1* may interact with* NM23* through* TIAM1* and* TP53*, which are associated with metastasis. We infer that* PSORS1C1* variants in HBV-related HCC patients result in immunosuppression and further cause the HBV to interfere with NM23 expression. However, how* PSORS1C1* affects NM23 expression remains unknown, and more experiments are needed to investigate the specific mechanism.

A correlation between* STARD3* and* NM23* expression was found in the GEO database.* STARD3* maps to chromosome 17q11-q12 and encodes a member of a subfamily of lipid trafficking proteins that are characterized by a C–terminal steroidogenic acute regulatory domain and an N–terminal metastatic lymph node 64 domain. Studies have reported that* STARD3* expression plays a role in focal adhesion kinase, and correlates with adhesive capacity and prognosis in breast cancer patients [36, 37]. The results of our pathway analysis showed that* NM23* and* STARD3* may be coexpressed. She et al. [38] showed that NM23 overexpression reduces the phosphorylation of focal adhesion kinase, mediating the invasive process of hepatocarcinoma cells. These may have a coordinated impact on modulating the cell adhesive capability via upregulated expression, due to mutual gene interactions. We also found that* STARD3* variants rs1877031 and rs11869286 were partly associated with NM23 expression. rs1877031 genotype TC has been reported to promote histogenesis in gastric cancer, and* STARD3 *haplotype CCCT (rs9972882, rs881844, rs11869286, and rs1877031) conferred a protective effect on susceptibility to gastric cancer [39]. In our haplotype analysis,* STARD3* (rs11869286, rs1877031) haplotype CA, GA, and CG had a lower cumulative effect on the expression of NM23 (OR=0.65, 95% CI=0.47–0.91), compared to haplotype GG. Moreover, STARD3 colocatizes with EGFR, which may influence clinical phenotypes associated with EGFR by affecting its expression and amplification [40]. Mandai et al. [41] reported that EGFR is correlated with NM23 expression. According to these studies,* STARD3* variants may play a role in regulating NM23 expression through candidate SNPs' LD effects and effects on EGFR expression, but the specific mechanisms require further investigation.

Several limitations of this study warrant discussion. First, our sample size is modest, as is common to many pharmacogenomic GWAS, and additional studies with larger sample sizes and multiple centers are needed to clarify our results. In addition, because the subjects evaluated in this study included minority subjects, racial heterogeneity may also represent a major limitation of the study. However, we accounted for this by including race, age, and gender as covariates in our GWAS model, and based on the low genomic inflation factor and the Q–Q plot, there is no evidence of population stratification. Finally, our research is preliminary, and further mechanistic and functional studies should be undertaken to discern the potential role of variants near* PSORS1C1* and* STARD3*.

In summary, we identified high NM23 mRNA level offering high diagnostic ability for the discrimination of HCC and demonstrated that genetic variants near* PSORS1C1* and* STARD3* are associated with NM23 expression in HBV-related HCC. Moreover, variants near* PSORS1C1 *(rs560052000, rs541820233, rs556285588, rs3095301, and rs3131003) are associated with the clinical outcomes acting as potential biomarkers for the prediction of postoperative patients with HBV-related HCC. The associations and molecular mechanisms of* NM23* regulation merit further research.

## Figures and Tables

**Table 1:** Clinicopathological characteristics of HBV-related HCC cases after data quality control.

	NM23							OS			RFS		
	—	1+	2+	3+	OR^*∗*^ (95% CI)	*P* value^†^	Total number	MST (months)	*P* value^‡^	HR_OS_^ ^^‡^ (95% CI)	MRT (months)	*P* value^‡^	HR_RFS_^ ^^‡^ (95% CI)
Gender						0.281			0.400			0.651	
Male	40	241	39	45	1.01 (0.53–1.95)		365	48		0.93 (0.51–1.69)	7		1.09 (0.73–1.63)
Female	3	31	7	2	Ref.		43	82		Ref.	12		Ref.
Age (years)						**0.017**			0.485			0.718	
≤46	20	144	35	24	1.45 (0.97–2.19)		223	61		0.87 (0.61–1.25)	7		1.05 (0.80–1.37)
>46	23	128	11	23	Ref.		185	45		Ref.	11		Ref.
Race						0.159			0.972			0.761	
Han	32	174	25	26	**0.62** **(0.41–0.95)**		257	48		0.92 (0.64–1.31)	7		0.96 (0.73–1.26)
Minority	11	98	21	21	Ref.		151	50		Ref.	11		Ref.
Preoperation TACE						0.359			0.640			0.592	
No	30	215	36	40	1.46 (0.88–2.40)		321	51		0.91 (0.61–1.37)	10		0.92 (0.67–1.27)
Yes	13	57	10	7	Ref.		87	44		Ref.	6		Ref.
Postoperation TACE						0.530			0.717			0.416	
No	26	156	25	22	0.76 (0.88–2.40)		229	47		0.94 (0.70–1.27)	7		0.90 (0.68–1.18)
Yes	17	116	21	25	Ref.		179	76		Ref.	13		Ref.
Smoking status						0.967			0.088			0.053	
None	14	99	16	17	1.04 (0.51–1.14)		146	39		1.24 (0.76–2.03)	5		1.31 (0.98–1.74)
Ever	29	173	30	30	Ref.		262	61		Ref.	12		Ref.
Drinking status						0.471			0.359			0.443	
None	16	107	19	24	1.32 (0.88–1.98)		166	44		0.82 (0.50–1.32)	6		1.11 (0.84–1.46)
Ever	27	165	27	23	Ref.		242	51		Ref.	11		Ref.
BMI						**0.012**			0.480			0.603	
≤25	35	206	39	45	**1.75** **(1.05–2.93)**		325	48		1.03 (0.67–1.59)	11		0.92 (0.66–1.29)
>25	8	66	7	2	Ref.		83	63		Ref.	6		Ref.
serum AFP (ng/ml)						0.099			0.101			0.353	
≤400	23	143	19	19	**0.61** **(0.40**–**0.93)**		204	61		1.00 (0.69–1.44)	13		0.88 (0.67–1.16)
>400	16	108	26	26	Ref.		176	41		Ref.	6		Ref.
NA	4	21	1	2			28						
Child–Pugh						0.509			**0.005**			**0.044**	
A	35	237	37	39	0.88 (0.50–1.54)		348	51		0.76 (0.48–1.20)	9		0.66 (0.43–1.01)
B	8	35	9	8	Ref.		60	31		Ref.	7		Ref.
BCLC stage						0.183			**1.35 × 10** ^**-10**^			**0.001**	
A	30	153	26	22	0.66 (0.41–1.05)		231	101		1.49 (0.40–5.56)	14		**0.57** **(0.42**–**0.77)**
B	4	46	10	6	0.92 (0.49–1.71)		66	39		1.92 (0.46–8.00)	6		0.66 (0.43–1.03)
C	9	73	10	19	Ref.		111	27		Ref.	3		Ref.
Number of tumors						0.247			**1.95 × 10** ^**-4**^			0.137	
Single(n=1)	36	202	32	31	0.63 (0.40–1.00)		301	58		0.71 (0.39–1.29)	11		0.80 (0.59–1.09)
Multiple(n>1)	7	70	14	16	Ref.		107	28		Ref.	5		Ref.
Tumor size (cm)						0.659			**0.038**			0.175	
≤10	37	222	36	36	0.72 (0.44–1.20)		331	51		0.66 (0.43–1.02)	12		0.81 (0.58–1.12)
>10	6	50	10	11	Ref.		77	40		Ref.	4		Ref.
Intrahepatic metastasis						0.325			**4.12 × 10** ^**-4**^			**6.03 × 10** ^**-5**^	
Absence	24	147	26	19	0.79 (0.53–1.18)		216	76		1.01 (0.67–1.52)	13		**0.59** **(0.45**–**0.78)**
Presence	19	125	20	28	Ref.		192	36		Ref.	4		Ref.
Distant metastasis						0.586^#^			0.051			0.178	
Absence	40	249	40	41	0.64 (0.33–1.24)		370	51		0.38 (0.11–1.34)	11		0.77 (0.51–1.15)
Presence	3	23	6	6	Ref.		38	32		Ref.	3		Ref.
Cirrhosis						0.246			0.567			0.923	
Absence	6	34	3	2	0.54 (0.28–1.04)		45	48		0.99 (0.59–1.65)	13		0.98 (0.62–1.54)
Presence	37	237	43	45	Ref.		362	48		Ref.	7		Ref.
NA	0	1	0	0			1						
Pathological grade						0.571			0.267			0.105	
Well	4	17	2	1	0.54 (0.22–1.30)		24	39		1.07 (0.57–2.02)	4		1.80 (0.84–3.85)
Moderately and poorly	35	222	39	36	Ref.		332	47		Ref.	7		Ref.
NA	4	33	5	10			52						
Regional invasion						**0.005**			0.407			0.215	
Absence	38	240	34	34	**0.41** **(0.24**–**0.71)**		346	51		0.81 (0.48–1.38)	11		0.81 (0.58–1.14)
Presence	5	32	12	13	Ref.		62	40		Ref.	3		Ref.
PVTT						0.162			**1.01 × 10** ^**-10**^			**6.75 × 10** ^**-5**^	
None	37	225	42	32	0.35 (0.10–1.25)		336	74		**0.10** **(0.03**–**0.44)**	12		**0.28** **(0.13**–**0.60)**
VP1	1	9	0	1	0.24 (0.04–1.42)		11	28		**0.27** **(0.08**–**0.99)**	2		0.37 (0.12–1.12)
VP2	0	11	1	5	0.93 (0.20–4.31)		17	18		0.53 (0.16–1.74)	2		0.64 (0.25–1.66)
VP3	4	23	2	6	0.39 (0.09–1.59)		35	18		0.36 (0.12–1.10)	3		0.49 (0.20–1.19)
VP4	1	4	1	3	Ref.		9	8		Ref.	2		Ref.
Radical resection						0.847			**0.025**			0.306	
No	24	147	29	28	1.14 (0.76–1.72)		228	73		1.01 (0.67–1.52)	12		0.87 (0.67–1.15)
Yes	18	114	17	19	Ref.		168	40		Ref.	6		Ref.
NA	1	11	0	0			12						
Antiviral therapies						**0.033**			**0.005**			0.800	
No	36	174	26	28	**0.59** **(0.39**–**0.90)**		264	41		**1.88** **(1.22**–**2.90)**	6		1.03 (0.79–1.36)
Yes	7	98	20	19	Ref.		144	NA (>45)		Ref.	13		Ref.
NM23 expression						NA			0.378			0.802	
Negative	43	-	-	-			43	47		1.12 (0.54–2.32)	14		0.88 (0.50–1.53)
1+	-	272	-	-			272	45		1.09 (0.62–1.92)	7		1.08 (0.72–1.61)
2+	-	-	46	-			46	73		0.68 (0.31–1.46)	7		0.99 (0.54–1.80)
3+	-	-	-	47			47	40		Ref.	11		Ref.

*Note*. ^*∗*^OR for univariate ordinal logistic regression analysis; when the *P*–value of the OR in the test of parallel lines was less than 0.05, multivariate logistic regression analysis was used but is not shown in the table. ^†^*P*–value for Chi-square test or Fisher's exact test. ^‡^HR and *P*–values are for univariate survival analysis. PVTT was classified using the radiographic results of enhanced computed tomography scanning as follows: vp1=PVTT in distal to second-order portal branches; vp2=PVTT in second–order portal branches; vp3=PVTT in first–order branches; and vp4=PVTT in the main trunk [15].

*Abbreviations*. GWAS: genome–wide association study; OR: odds ratio; 95% CI: 95% confidence intervals; MST: median survival time; MRT: median recurrence time; HR, hazard ratio; Ref.: reference; NA: not applicable; TACE: transcatheter arterial chemoembolization; BMI: body mass index; AFP: alpha–fetoprotein; BCLC: Barcelona Clinic Liver Cancer; PVTT: portal vein tumor thrombus.

**Table 2:** Association between haplotypes of *PSORS1C1* and *STARD3* and NM23 expression.

Haplotypes	NM23				Ad OR^★^	*P*–value	Ad HR_OS_^ ^^†^	*P*–value	Ad HR_RFS_^ ^^†^^ ^	*P*–value
	—	1+	2+	3+	(95% CI)		(95% CI)		(95% CI)	
PSORS1C1										
TGCACA	40	273	61	63	Ref.		Ref.		Ref.	
CAGGTG	37	171	19	20	0.44 (0.3 0–0.65)	**2.70 × 10** ^**-5**^	0.71 (0.42–1.22)	0.219	0.87 (0.46–1.63)	0.653
CAGACA	6	64	9	5	0.56 (0.32–0.99)	**0.048**	0.99 (0.65–1.51)	0.967	1.66 (0.95–2.90)	0.073
Others^a^	3	36	3	6	0.60 (0.3 0–1.18)	0.135	0.97 (0.74–1.27)	0.805	2.04 (0.99–4.20)	0.054
All others^b^	46	271	31	31	0.48 (0.35–0.68)	**2.05 × 10** ^**-5**^	0.93 (0.73–1.18)	0.561	0.74 (0.50–1.10)	0.136
STARD3										
GG	46	329	58	69	Ref.		Ref.			
Others^c^	40	209	34	25	0.65 (0.47–0.91)	**0.012**	0.97 (0.76–1.24)	0.814	1.18 (0.74–1.88)	0.498

*Note*. ^★^OR, ^†^HR and *P*–value are adjusted for age, gender, race, smoking status, drinking status, BMI, BCLC stage, Child–Pugh stage, preoperative serum AFP level, TACE status before hepatectomy, pathological grade, cirrhosis, intrahepatic metastasis, PVTT, regional invasion, radical resection, and use of antiviral therapies. ^a^Others include CGCACA and CGGGCA. ^b^The group of all others is merged CAGGTG, CAGACA, CGCACA, and CGGGCA. ^c^Others include CA, GA, CG, and the total number of patients harbour GA and CG is 6.

*Abbreviations*. OR: odds ratio; HR: hazard ratio; 95% CI: 95% confidence intervals; Ad: adjusted.

## Data Availability

All the data supporting our findings can be found in the Results and Supplementary materials section of the paper. Please contact corresponding author for more data on reasonable request.

## Supplementary Materials

Supplementary Materials 
** Supplementary Table S1**. Primers of polymerase chain reaction for candidate SNPs.** Supplementary Table S2**. NM23 expression and ROC analysis of enrolled HCC datasets in this study.** Supplementary Table S3**. Candidate SNPs in HBV-related HCC patients.** Supplementary Table S4**. Association between genotypes of candidate SNPs with NM23 expression.** Supplementary Table S5**. The association between Gene expression levels and clinical outcomes of HBV-related HCC patients.** Supplementary Table S6**. Association between* PSORS1C1* and* STARD3* SNPs with clinical outcomes of HBV-related HCC patients after hepatic resection.** Supplementary Figure S1. **Candidate SNPs eQTL analysis in GTEx portal.** Supplementary Figure S2. **Stratified analysis on association of rs541820233–TC (**A**), rs556285588–AG (**B**), and rs560052000–GC (**C**) with outcomes in HBV-related HCC patients. The HRs are indicated for overall survival. The figure is stratified by favorable and adverse strata.
