# Supplementary material for: Clinical Implication and the Hereditary Factors of NM23 in Hepatocellular Carcinoma Based on Bioinformatics Analysis and Genome–Wide Association Study
Source: J Oncol. 2018 Dec 18;2018:6594169. doi: 10.1155/2018/6594169 (PMC6312618; doi:10.1155/2018/6594169)
Supplement: Supplementary Materials — Supplementary Table S1. Primers of polymerase chain reaction for candidate SNPs. Supplementary Table S2. NM23 expression and ROC analysis of enrolled HCC datasets in this study. Supplementary Table S3. Candidate SNPs in HBV-related HCC patients. Supplementary Table S4. Association between genotypes of candidate SNPs with NM23 expression. Supplementary Table S5. The association between Gene expression levels and clinical outcomes of HBV-related HCC patients. Supplementary Table S6. Association between PSORS1C1 and STARD3 SNPs with clinical outcomes of HBV-related HCC patients after hepatic resection. Supplementary Figure S1. Candidate SNPs eQTL analysis in GTEx portal. Supplementary Figure S2. Stratified analysis on association of rs541820233–TC (A), rs556285588–AG (B), and rs560052000–GC (C) with outcomes in HBV-related HCC patients. The HRs are indicated for overall survival. The figure is stratified by favorable and adverse strata. [file 6594169.f1.zip › Supplementary material-Tables_JO_2602127.docx]

**Supplementary Table S1. Primers of polymerase chain reaction for candidate SNPs**

| SNPs | Primers | Sequences (5' to 3') | Annealing  temperature (℃) | Amplification  length (bp) |
| --- | --- | --- | --- | --- |
| rs541820233 | Forward | CGCTATTCCCTCAGACA | 58 | 434 |
|  | Reverse | GACTACAGGTGCCCATC |  |  |
| rs556285588 | Forward | CACAGGTGGGCAGGACA | 59 | 511 |
|  | Reverse | AGGGAAACTCCGTCTCAA |  |  |
| rs560052000 | Forward | CACAGGTGGGCAGGACA | 62 | 510 |
|  | Reverse | GGGAAACTCCGTCTCAAAA |  |  |
| rs3095302 | Forward | TCGGAACACCTGATTGG | 62 | 584 |
|  | Reverse | CCTGGGTGACAGAGTGAGA |  |  |
| rs3095301 | Forward | AATCCCTCCAGCACCTAC | 64 | 705 |
|  | Reverse | GCCACAACAACACCCTC |  |  |
| rs3131003 | Forward | TCTGCGAAGACTGACCCT | 61 | 512 |
|  | Reverse | GAATCCCTCCAGCACCTA |  |  |
| rs11869286 | Forward | CTTCGGGAGCCATCAGT | 62 | 555 |
|  | Reverse | AGCAAGGCAGTCACAGGA |  |  |
| rs1877031 | Forward | GCAGAGGCTGGGAAGTG | 60 | 519 |
|  | Reverse | AGGCTGAGGCAGGAGAA |  |  |

**Supplementary Table S2. NM23 expression and ROC of enrolled HCC datasets in this study.**

| **First author** | **Country** | **Year** | **Dataset** | **Platform** | **No.** | **Non-tumor** | | **No.** | **HCC** | | **AUC** | **95%CI** | |
| --- | --- | --- | --- | --- | --- | --- | --- | --- | --- | --- | --- | --- | --- |
|  |  |  |  |  |  | Mean | SD |  | Mean | SD |  | lower | upper |
| Hoshida Y [1] | USA | 2008 | GEO:GSE10143 | Illumina GPL5474 | 307 | 15675.52 | 9906.90 | 80 | 36980.98 | 7930.51 | 0.933 | 0.909 | 0.957 |
| Satow R [2] | Japan | 2010 | GEO:GSE12941 | Affymetrix GPL5175 | 10 | 7.93 | 0.19 | 10 | 8.91 | 0.50 | 1 | 1 | 1 |
| Tildiz G [3] | Turkey | 2013 | GEO:GSE17548 | Affymetrix GPL570 | 20 | 9.27 | 0.54 | 17 | 10.16 | 0.79 | 0.812 | 0.661 | 0.963 |
| Archer KJ [4] | USA | 2009 | GEO:GSE17967 | Affymetrix GPL571 | 47 | 7.56 | 0.41 | 16 | 7.61 | 0.33 | 0.535 | 0.375 | 0.694 |
| Zhang HH | USA | 2014 | GEO:GSE22405 | Affymetrix GPL10553 | 24 | 7.75 | 0.78 | 24 | 8.61 | 1.07 | 0.733 | 0.588 | 0.878 |
| Yang F [5] | China | 2011 | GEO:GSE27462 | Arraystar GPL11269 | 5 | 3280.61 | 2277.56 | 5 | 6912.24 | 2148.63 | 0.840 | 0.580 | 1 |
| Lim HY [6] | South Korea | 2013 | GEO:GSE36376 | Illumina GPL10558 | 193 | 10.26 | 0.35 | 240 | 11.52 | 0.61 | 0.969 | 0.953 | 0.986 |
| Kim JH [7] | USA | 2014 | GEO:GSE39791 | Illumina GPL10558 | 72 | 9.81 | 0.45 | 72 | 10.63 | 0.66 | 0.846 | 0.781 | 0.912 |
| Ueda T [8] | Japan | 2013 | GEO:GSE44074 | Kanazawa GPL13536 | 71 | 0.26 | 0.37 | 34 | 0.74 | 0.53 | 0.772 | 0.665 | 0.879 |
| Wei L [9] | China | 2014 | GEO:GSE45114 | CapitalBio GPL5918 | 13 | 0.02 | 0.45 | 14 | 0.96 | 0.60 | 0.898 | 0.811 | 0.985 |
| Chen YL [10] | China | 2013 | GEO:GSE46408 | AGilent GPL4133 | 6 | 13659.09 | 3576.94 | 6 | 38329.65 | 8632.18 | 1 | 1 | 1 |
| Kojima K [11] | USA | 2014 | GEO:GSE46444 | Illumina GPL13369 | 48 | 1106.74 | 822.95 | 88 | 1807.71 | 1161.19 | 0.687 | 0.596 | 0.779 |
| Wang K [12] | China | 2015 | GEO:GSE49713 | Arraystar GPL11269 | 5 | 177.42 | 89.60 | 5 | 595.41 | 135 | 1 | 1 | 1 |
| Neumann O [13] | Germany | 2012 | GEO:GSE50579 | AGilent GPL14550 | 10 | 12.31 | 0.49 | 67 | 13.26 | 0.55 | 0.907 | 0.801 | 1 |
| Villa E [14] | Italy | 2016 | GEO:GSE54236 | AGilent GPL6480 | 80 | 12.53 | 0.38 | 81 | 13.02 | 0.55 | 0.761 | 0.687 | 0.835 |
| Yuan SX [15] | USA | 2016 | GEO:GSE54238 | Arraystar GPL16955 | 30 | 559.12 | 864.64 | 26 | 1076.57 | 943.79 | 0.815 | 0.688 | 0.943 |
| Melis M [16] | USA | 2014 | GEO:GSE55092 | Affymetrix GPL570 | 91 | 8.80 | 0.63 | 49 | 9.96 | 0.92 | 0.859 | 0.789 | 0.929 |
| Villanueva A [17] | USA | 2011 | GEO:GSE56140 | Illumina GPL18461 | 34 | 1861.53 | 635.31 | 35 | 3998.18 | 1163.03 | 0.927 | 0.855 | 0.999 |
| Mah WC [18] | Singapore | 2014 | GEO:GSE57957 | Illumina GPL10558 | 39 | 3126.19 | 1065.95 | 39 | 6631.62 | 2641 | 0.911 | 0.840 | 0.981 |
| Udali S [19] | Italy | 2015 | GEO:GSE59259 | Nimble Gen GPL18451 | 8 | 4230.61 | 836.11 | 8 | 8475.5 | 2513.15 | 0.984 | 0.936 | 1 |
| Wang YH [20] | China | 2014 | GEO:GSE60502 | Affymetrix GPL96 | 18 | 10.39 | 0.30 | 18 | 11.15 | 0.60 | 0.923 | 0.838 | 1 |
| Schulze K [21] | France | 2015 | GEO:GSE62232 | Affymetrix GPL570 | 10 | 338.40 | 84.96 | 81 | 895.31 | 414.20 | 0.967 | 0.932 | 1 |
| Makowska Z [22] | Switzerland | 2016 | GEO:GSE64041 | Affymetrix GPL6244 | 65 | 8.75 | 0.23 | 60 | 9.31 | 0.41 | 0.889 | 0.830 | 0.949 |
| Tao Y | China | 2015 | GEO:GSE74656 | Affymetrix GPL16043 | 5 | 8.80 | 0.42 | 5 | 10.43 | 0.72 | 0.96 | 0.843 | 1 |
| Grinchuk OV [23] | Singapore | 2018 | GEO:GSE76427 | Illumina GPL10558 | 52 | 3747.04 | 1179.14 | 115 | 6582.22 | 2534.92 | 0.874 | 0.820 | 0.927 |
| Tu X | China | 2017 | GEO:GSE84005 | Affymetrix GPL5175 | 38 | 8.39 | 0.41 | 38 | 9.60 | 0.64 | 0.943 | 0.890 | 0.996 |
| Wurmbach E [24] | USA | 2007 | Oncomine:WurmbachLiver | Affymetrix GPL570 | 40 | 616.49 | 355.49 | 35 | 1162.83 | 709.52 | 0.784 | 0.680 | 0.888 |
| Mas VR [25] | USA | 2009 | Oncomine:MasLiver | Affymetrix GPL571 | 77 | 8.42 | 0.58 | 38 | 8.23 | 0.69 | 0.425 | 0.319 | 0.531 |

**Abbreviations**: HCC, hepatocellular carcinoma; SD, standard deviation; AUC, area under the curve; CI, confidence interval.

**Supplementary Table S3. Candidate SNPs in HBV-related HCC patients.**

| SNPs | CHr | Position | Gene | Allele | Function | MAF | HWE | *P*-value |
| --- | --- | --- | --- | --- | --- | --- | --- | --- |
| rs541820233 | 6 | 31087354 | PSORS1C1 | C/T | Intron | 0.46 | 0.833 | 5.37×10^-4^ |
| rs556285588 | 6 | 31090672 | PSORS1C1 | A/G | Intron | 0.42 | 0.599 | 8.40×10^-4^ |
| rs560052000 | 6 | 31090837 | PSORS1C1 | G/C | Intron | 0.43 | 0.561 | 4.55×10^-4^ |
| rs3095302 | 6 | 31093066 | PSORS1C1 | G/A | Intron | 0.33 | 0.57 | 3.16×10^-3^ |
| rs3095301 | 6 | 31093356 | PSORS1C1 | T/C | Intron | 0.31 | 0.741 | 3.08×10^-3^ |
| rs3131003 | 6 | 31093482 | PSORS1C1 | G/A | Utr5 | 0.31 | 0.949 | 1.04×10^-3^ |
| rs11869286 | 17 | 37813856 | STARD3 | G/C | Intron | 0.38 | 0.724 | 5.67×10^-3^ |
| rs1877031 | 17 | 37814080 | STARD3 | G/A | Nonsynonymous | 0.38 | 0.974 | 4.99×10^-3^ |

**Abbreviations**: SNP, single nucleotide polymorphism; Chr, chromosome; MAF, minor allele frequency; HWE, Hardy–Weinberg equilibrium.

**Supplementary Table S4. Association between genotypes of candidate SNPs with NM23 expression.**

|  | NM23 | | | |  |  |  |  |
| --- | --- | --- | --- | --- | --- | --- | --- | --- |
| SNPs | — | 1+ | 2+ | 3+ | OR(95%CI)^★^ | *P*-value^★^ | Ad OR(95%CI)^★★^ | *P*-value^★★^ |
| rs541820233 |  |  |  |  |  |  |  |  |
| TT | 9 | 65 | 17 | 22 | Ref. |  | Ref. |  |
| TC | 22 | 143 | 27 | 19 | 0.54(0.34–0.86) | **0.010** | 0.44(0.26–0.77) | **0.003** |
| CC | 12 | 64 | 2 | 6 | 0.30(0.16–0.55) | **9.30×10^-5^** | 0.21(0.10–0.43) | **1.80×10^-5^** |
| rs556285588 |  |  |  |  |  |  |  |  |
| AA | 10 | 54 | 1 | 3 | Ref. |  | Ref. |  |
| AG | 23 | 133 | 26 | 21 | 2.17(1.20–3.94) | **0.011** | 2.49(1.24–5.02) | **0.011** |
| GG | 10 | 85 | 19 | 23 | 3.41(1.81–6.42) | **1.42×10^-4^** | 4.21(2.01–8.79) | **1.32×10^-4^** |
| rs560052000 |  |  |  |  |  |  |  |  |
| CC | 9 | 78 | 19 | 23 | Ref. |  | Ref. |  |
| CG | 23 | 136 | 25 | 20 | 0.56(0.39–0.89) | **0.013** | 0.50(0.30–0.86) | **0.011** |
| GG | 11 | 58 | 2 | 4 | 0.29(0.16–0.54) | **8.80×10^-5^** | 0.23(0.11–0.47) | **6.88×10^-5^** |
| rs3095302 |  |  |  |  |  |  |  |  |
| AA | 14 | 112 | 27 | 28 | Ref. |  | Ref. |  |
| AG | 19 | 132 | 18 | 15 | 0.56(0.36–0.86) | **0.008** | 0.53(0.32–0.87) | **0.012** |
| GG | 10 | 28 | 1 | 4 | 0.27(0.13–0.55) | **3.53×10^-4^** | 0.22(0.09–0.53) | **0.001** |
| rs3095301 |  |  |  |  |  |  |  |  |
| TT | 9 | 24 | 0 | 3 | Ref. |  | Ref. |  |
| TC | 20 | 131 | 19 | 16 | 2.56(1.20–5.48) | **0.015** | 3.01(1.21–7.49) | **0.018** |
| CC | 14 | 117 | 27 | 28 | 4.40(2.03–9.51) | **1.68×10^-4^** | 5.47(2.17–13.76) | **3.07×10^-4^** |
| rs3131003 |  |  |  |  |  |  |  |  |
| AA | 14 | 119 | 27 | 30 | Ref. |  | Ref. |  |
| AG | 20 | 129 | 19 | 14 | 0.54(0.35–0.84) | **0.005** | 0.50(0.30–0.82) | **0.006** |
| GG | 9 | 24 | 0 | 3 | 0.22(0.10–0.48) | **1.20×10^-4^** | 0.17(0.07–0.44) | **2.00×10^-4^** |
| rs11869286 |  |  |  |  |  |  |  |  |
| GG | 11 | 105 | 19 | 24 | Ref. |  | Ref. |  |
| GC | 24 | 123 | 20 | 21 | 0.67(0.43–1.03) | 0.068 | 0.75(0.45–1.23) | 0.254 |
| CC | 8 | 44 | 7 | 2 | 0.48(0.26–0.91) | **0.023** | 0.41(0.20–0.86) | **0.018** |
| rs1877031 |  |  |  |  |  |  |  |  |
| AA | 8 | 42 | 7 | 2 | Ref. |  | Ref. |  |
| AG | 24 | 129 | 20 | 21 | 1.36(0.73–2.52) | 0.334 | 2.47(1.19–5.14) | **0.016** |
| GG | 11 | 101 | 19 | 24 | 2.11(1.12–3.99) | **0.021** | 1.79(0.88–3.56) | 0.106 |

**Note**:^★^OR (95%CI) and *P*-value are for univariate ordinal logistic regression analysis. ^★★^ OR (95%CI) and *P*-value are adjusted for age, gender, race, smoking status, drinking status, BMI, BCLC stage, Child-Pugh stage, preoperative serum AFP level, TACE status before hepatectomy, pathological grade, cirrhosis, intrahepatic metastasis, PVTT, regional invasion, radical resection, and use of antiviral therapies.

**Abbreviations**: SNP, single nucleotide polymorphism; OR, odds ratio; 95% CI, 95% confidence intervals; Ad OR, adjusted OR; Ref., reference.

**Supplementary Table S5. The association between Gene expression levels and clinical outcomes of HBV-related HCC patients.**

|  |  |  | OS | | | |  | RFS | | | |  |
| --- | --- | --- | --- | --- | --- | --- | --- | --- | --- | --- | --- | --- |
| Gene |  | number | MST | *P*-value | HR^★^ | 95%CI^★^ | *P*-value | MRT | *P*-value | HR^★^ | 96%CI^★^ | *P*-value |
| NM23 | low | 106 | >67.3 | 0.149 | Ref. |  | 0.579 | **59.5** | **0.011** | Ref. |  | **0.044** |
|  | high | 106 | 60.5 |  | 1.14 | 0.73-1.77 |  | **29.9** |  | **1.45** | **1.01-2.13** |  |
| STARD3 | low | 106 | >67.0 | 0.242 | Ref. |  | 0.411 | 36.6 | 0.736 | Ref. |  | 0.984 |
|  | high | 106 | >67.4 |  | 0.83 | 0.53-1.29 |  | 48.0 |  | 1.00 | 0.69-1.45 |  |
| PSORS1C1 | low | 106 | >67.4 | 0.480 | Ref. |  | 0.606 | 51.1 | 0.434 | Ref. |  | 0.360 |
|  | high | 106 | >67.3 |  | 1.12 | 0.73-1.74 |  | 36.6 |  | 1.12 | 0.82-1.71 |  |

Note: ^★^HRs are adjusted for age, gender, Barcelona Clinic Liver Cancer stage, cirrhosis

**Abbreviations**: OS, overall survival; RFS, recurrence-free survival; MST, median survival time; MRT, median recurrence time; HR, hazard ratio; 95% CI, 95% confidence intervals; Ref, reference.

**Supplementary Table S6. Association between *PSORS1C1* and *STARD3* SNPs with clinical outcomes of HBV-related HCC patients after hepatic resection.**

|  |  |  | OS | |  |  |  |  | RFS | |  |  |  |  |
| --- | --- | --- | --- | --- | --- | --- | --- | --- | --- | --- | --- | --- | --- | --- |
| SNPs |  | Number | MST | *P*-value | HR_OS_^★^  (95%CI) | *P*-value^★^ | Ad HR_OS_^★★^  (95%CI) | *P*-value^★★^ | MRT | *P*-value | HR_RFS_^★^  (95%CI) | *P*-value^★^ | Ad HR_RFS_^★★^  (95%CI) | *P*-value^★★^ |
|  |  |  | (months) |  |  |  |  |  | (months) |  |  |  |  |  |
| rs541820233 | TT | 118 | 40 | **0.007** | Ref. | **0.007** | Ref. | **0.017** | 7 | 0.069 | Ref. | 0.088 | Ref. | 0.077 |
|  | TC | 204 | 73 |  | 0.63  (0.43–0.92) | **0.015** | 0.64  (0.45–0.90) | **0.012** | 12 |  | 0.81  (0.59–1.11) | 0.190 | 0.73  (0.50–1.07) | 0.102 |
|  | CC | 86 | 35 |  | 1 .00  (0.68–1.49) | 0.984 | 0.99  (0.65–1.51) | 0.970 | 6 |  | 1.17  (0.80–1.71) | 0.420 | 1.12  (0.71–1.77) | 0.617 |
|  | TT+CC *vs* TC^a^ | 204 | 40 | **0.002** | 0.63  ( 0.47–0.84) | **0.002** | 0.64  (0.47–0.87) | **0.004** | 6 | **0.034** | 0.76  (0.58–0.99) | **0.042** | 0.69  (0.50–0.96) | **0.028** |
| rs556285588 | AA | 70 | 35 | **0.016** | Ref. | **0.014** | Ref. | **0.013** | 6 | 0.531 | Ref. | 0.560 | Ref. | 0.713 |
|  | AG | 197 | 73 |  | 0.61  (0.41–0.92) | **0.019** | 0.58  (0.38–0.90) | **0.015** | 12 |  | 0.84  (0.58–1.22) | 0.356 | 0.83  (0.53–1.30) | 0.413 |
|  | GG | 141 | 41 |  | 0.93  (0.62–1.41) | 0.744 | 0.92  (0.59–1.41) | 0.692 | 7 |  | 0.81  (0.55–1.20) | 0.299 | 0.85  (0.53–1.37) | 0.515 |
|  | AA+GG *vs* AG^b^ | 211 | 40 | **0.004** | 0.64  (0.48–0.87) | **0.004** | 0.62  (0.45–0.86) | **0.004** | 7 | 0.797 | 0.97  (0.74–1.26) | 0.805 | 0.93  (0.68–1.26). | 0.623 |
| rs560052000 | CC | 133 | 40 | **0.003** | Ref. | **0.005** | Ref. | **0.010** | 7 | 0.078 | Ref. | 0.097 | Ref. | 0.054 |
|  | CG | 198 | 76 |  | 0.62  (0.45–0.87) | **0.005** | 0.61  (0.43–0.87) | **0.006** | 13 |  | 0.80  (0.59–1.08) | 0.142 | 0.68  (0.47–0.98) | **0.037** |
|  | GG | 77 | 34 |  | 1.05  (0.71–1.55) | 0.826 | 1.00  ( 0.65–1.54) | 0.995 | 6 |  | 1.15  (0.78–1.68) | 0.484 | 1.05  (0.66–1.65) | 0.851 |
|  | CC+GG *vs* CG^c^ | 210 | 40 | **0.001** | 0.61  ( 0.46–0.82) | **0.001** | 0.61  ( 0.44–0.84) | **0.002** | 6 | **0.034** | 0.76  (0.58–0.99) | **0.043** | 0.67  (0.48–0.93) | **0.016** |
| rs3095301 | TT | 37 | 28 | **0.043** | Ref. | 0.056 | Ref. | 0.166 | 6 | 0.439 | Ref. | 0.469 | Ref. | 0.246 |
|  | TC | 178 | 52 |  | 0.56  (0.35–0.90) | **0.017** | 0.62  (0.37–1.03) | 0.063 | 11 |  | 0.83  (0.49–1.41) | 0.491 | 1.14  (0.60–2.17) | 0.687 |
|  | CC | 193 | 45 |  | 0.67  (0.42–1.07) | 0.093 | 0.73  (0.44–1.20) | 0.219 | 7 |  | 0.98  (0.58–1.67) | 0.938 | 1.48  (0.77–2.85) | 0.238 |
|  | TT+CC *vs* TC^d^ | 230 | 44 | 0.097 | 0.78  (0.58–1.05) | 0.100 | 0.80  (0.58–1.10) | 0.161 | 7 | 0.200 | 0.85  (0.65–1.11) | 0.220 | 0.82  (0.59–1.15) | 0.249 |
|  | CC+TC *vs* TT^e^ | 371 | 51 | **0.030** | 1.62  (1.04–2.54) | **0.033** | 0.87  (0.50–1.54) | 0.636 | 9 | 0.248 | 0.90  (0.54–1.50) | 0.678 | 1.27  (0.68–2.38) | 0.449 |
| rs3131003 | AA | 197 | 58 | 0.071 | Ref. | 0.106 | Ref. | 0.318 | 9 | 0.560 | Ref. | 0.587 | Ref. | 0.319 |
|  | AG | 174 | 51 |  | 0.92  (0.67–1.25) | 0.586 | 0.94  (0.68–1.31) | 0.731 | 11 |  | 0.87  (0.66–1.15) | 0.343 | 0.80  (0.56–1.12) | 0.193 |
|  | GG | 37 | 28 |  | 1.53  (0.96–2.45) | 0.073 | 1.39  ( 0.85–2.29) | 0.191 | 6 |  | 1.04  (0.61–1.77) | 0.885 | 0.69  (0.36–1.33) | 0.265 |
|  | AA+GG *vs* AG^f^ | 234 | 45 | 0.196 | 0.86  (0.64–1.15) | 0.299 | 0.89  (0.65–1.22) | 0.475 | 7 | 0.287 | 0.87  (0.67–1.14) | 0.307 | 0.85  (0.61–1.18) | 0.323 |
|  | AA+AG *vs* GG^g^ | 371 | 51 | **0.030** | 1.62  (1.04–2.54) | **0.033** | 1.15  (0.65–2.02) | 0.636 | 10 | 0.664 | 1.12  (0.67–1.86) | 0.678 | 0.79  (0.42–1.47) | 0.449 |

**Note**:^★^HR (95%CI) and *P*-value for univariate Cox proportional hazard regression model.  ^★★^HR (95%CI) and *P*-value are adjusted for age, gender, race, smoking status, drinking status, BMI, BCLC stage, Child-Pugh stage, preoperative serum AFP level, TACE status before hepatectomy, pathological grade, cirrhosis, intrahepatic metastasis, PVTT, regional invasion, radical resection, and use of antiviral therapies. ^a, b, c, d, e, f, g^These groups are merged genotypes regarded as the reference.

**Abbreviations**: SNPs, single nucleotide polymorphisms, OS, overall survival; RFS, recurrence free survival; HR, hazard ratio; 95% CI, 95% confidence intervals; MST, median survival time; MRT, median recurrence time; Ad HR, adjusted hazard ratio; Ref., reference.

**Supplementary material references**

1. Hoshida, Y.; Villanueva, A.; Kobayashi, M.; Peix, J.; Chiang, D.Y.; Camargo, A.; Gupta, S.; Moore, J.; Wrobel, M.J.; Lerner, J. Gene expression in fixed tissues and outcome in hepatocellular carcinoma. *New England Journal of Medicine* **2008**, *359*, 1995-2004.

2. Satow, R.; Shitashige, M.; Kanai, Y.; Takeshita, F.; Ojima, H.; Jigami, T.; Honda, K.; Kosuge, T.; Ochiya, T.; Hirohashi, S. Combined functional genome survey of therapeutic targets for hepatocellular carcinoma. *Clinical Cancer Research* **2010**, 1078-0432. CCR-1009-2214.

3. Yildiz, G.; Arslan-Ergul, A.; Bagislar, S.; Konu, O.; Yuzugullu, H.; Gursoy-Yuzugullu, O.; Ozturk, N.; Ozen, C.; Ozdag, H.; Erdal, E. Genome-wide transcriptional reorganization associated with senescence-to-immortality switch during human hepatocellular carcinogenesis. *PloS one* **2013**, *8*, e64016.

4. Archer, K.J.; Mas, V.R.; David, K.; Maluf, D.G.; Bornstein, K.; Fisher, R.A. Identifying genes for establishing a multigenic test for hepatocellular carcinoma surveillance in hepatitis c virus-positive cirrhotic patients. *Cancer Epidemiology and Prevention Biomarkers* **2009**, *18*, 2929-2932.

5. Yang, F.; Zhang, L.; Huo, X.s.; Yuan, J.h.; Xu, D.; Yuan, S.x.; Zhu, N.; Zhou, W.p.; Yang, G.s.; Wang, Y.z. Long noncoding rna high expression in hepatocellular carcinoma facilitates tumor growth through enhancer of zeste homolog 2 in humans. *Hepatology* **2011**, *54*, 1679-1689.

6. Lim, H.-Y.; Sohn, I.; Deng, S.; Lee, J.; Jung, S.H.; Mao, M.; Xu, J.; Wang, K.; Shi, S.; Joh, J.W. Prediction of disease-free survival in hepatocellular carcinoma by gene expression profiling. *Annals of surgical oncology* **2013**, *20*, 3747-3753.

7. Kim, J.H.; Sohn, B.H.; Lee, H.-S.; Kim, S.-B.; Yoo, J.E.; Park, Y.-Y.; Jeong, W.; Lee, S.S.; Park, E.S.; Kaseb, A. Genomic predictors for recurrence patterns of hepatocellular carcinoma: Model derivation and validation. *PLoS medicine* **2014**, *11*, e1001770.

8. Ueda, T.; Honda, M.; Horimoto, K.; Aburatani, S.; Saito, S.; Yamashita, T.; Sakai, Y.; Nakamura, M.; Takatori, H.; Sunagozaka, H. Gene expression profiling of hepatitis b-and hepatitis c-related hepatocellular carcinoma using graphical gaussian modeling. *Genomics* **2013**, *101*, 238-248.

9. Wei, L.; Lian, B.; Zhang, Y.; Li, W.; Gu, J.; He, X.; Xie, L. In *Application of microrna and mrna expression profiling on prognostic biomarker discovery for hepatocellular carcinoma*, Bmc Genomics, 2014; BioMed Central: p S13.

10. Chen, Y.-L.; Wang, T.-H.; Hsu, H.-C.; Yuan, R.-H.; Jeng, Y.-M. Overexpression of cthrc1 in hepatocellular carcinoma promotes tumor invasion and predicts poor prognosis. *PloS one* **2013**, *8*, e70324.

11. Kojima, K.; April, C.; Canasto-Chibuque, C.; Chen, X.; Deshmukh, M.; Venkatesh, A.; Tan, P.S.; Kobayashi, M.; Kumada, H.; Fan, J.-B. Transcriptome profiling of archived sectioned formalin-fixed paraffin-embedded (as-ffpe) tissue for disease classification. *PloS one* **2014**, *9*, e86961.

12. Wang, K.; xing Guo, W.; Li, N.; fang Gao, C.; Shi, J.; fu Tang, Y.; Shen, F.; chao Wu, M.; rong Liu, S.; qun Cheng, S. Serum lncrnas profiles serve as novel potential biomarkers for the diagnosis of hbv-positive hepatocellular carcinoma. *PloS one* **2015**, *10*, e0144934.

13. Neumann, O.; Kesselmeier, M.; Geffers, R.; Pellegrino, R.; Radlwimmer, B.; Hoffmann, K.; Ehemann, V.; Schemmer, P.; Schirmacher, P.; Bermejo, J.L. Methylome analysis and integrative profiling of human hccs identify novel protumorigenic factors. *Hepatology* **2012**, *56*, 1817-1827.

14. Villa, E.; Critelli, R.; Lei, B.; Marzocchi, G.; Cammà, C.; Giannelli, G.; Pontisso, P.; Cabibbo, G.; Enea, M.; Colopi, S. Neoangiogenesis-related genes are hallmarks of fast-growing hepatocellular carcinomas and worst survival. Results from a prospective study. *Gut* **2015**, gutjnl-2014-308483.

15. Yuan, S.x.; Wang, J.; Yang, F.; Tao, Q.f.; Zhang, J.; Wang, L.l.; Yang, Y.; Liu, H.; Wang, Z.g.; Xu, Q.g. Long noncoding rna dancr increases stemness features of hepatocellular carcinoma by derepression of ctnnb1. *Hepatology* **2016**, *63*, 499-511.

16. Melis, M.; Diaz, G.; Kleiner, D.E.; Zamboni, F.; Kabat, J.; Lai, J.; Mogavero, G.; Tice, A.; Engle, R.E.; Becker, S. Viral expression and molecular profiling in liver tissue versus microdissected hepatocytes in hepatitis b virus-associated hepatocellular carcinoma. *Journal of translational medicine* **2014**, *12*, 230.

17. Villanueva, A.; Hoshida, Y.; Battiston, C.; Tovar, V.; Sia, D.; Alsinet, C.; Cornella, H.; Liberzon, A.; Kobayashi, M.; Kumada, H. Combining clinical, pathology, and gene expression data to predict recurrence of hepatocellular carcinoma. *Gastroenterology* **2011**, *140*, 1501-1512. e1502.

18. Mah, W.-C.; Thurnherr, T.; Chow, P.K.; Chung, A.Y.; Ooi, L.L.; Toh, H.C.; Teh, B.T.; Saunthararajah, Y.; Lee, C.G. Methylation profiles reveal distinct subgroup of hepatocellular carcinoma patients with poor prognosis. *PLoS One* **2014**, *9*, e104158.

19. Udali, S.; Guarini, P.; Ruzzenente, A.; Ferrarini, A.; Guglielmi, A.; Lotto, V.; Tononi, P.; Pattini, P.; Moruzzi, S.; Campagnaro, T. DNA methylation and gene expression profiles show novel regulatory pathways in hepatocellular carcinoma. *Clinical epigenetics* **2015**, *7*, 43.

20. Wang, Y.-H.; Cheng, T.-Y.; Chen, T.-Y.; Chang, K.-M.; Chuang, V.P.; Kao, K.-J. Plasmalemmal vesicle associated protein (plvap) as a therapeutic target for treatment of hepatocellular carcinoma. *BMC cancer* **2014**, *14*, 815.

21. Schulze, K.; Imbeaud, S.; Letouzé, E.; Alexandrov, L.B.; Calderaro, J.; Rebouissou, S.; Couchy, G.; Meiller, C.; Shinde, J.; Soysouvanh, F. Exome sequencing of hepatocellular carcinomas identifies new mutational signatures and potential therapeutic targets. *Nature genetics* **2015**, *47*, 505.

22. Makowska, Z.; Boldanova, T.; Adametz, D.; Quagliata, L.; Vogt, J.E.; Dill, M.T.; Matter, M.S.; Roth, V.; Terracciano, L.; Heim, M.H. Gene expression analysis of biopsy samples reveals critical limitations of transcriptome‐based molecular classifications of hepatocellular carcinoma. *The Journal of Pathology: Clinical Research* **2016**, *2*, 80-92.

23. Grinchuk, O.V.; Yenamandra, S.P.; Iyer, R.; Singh, M.; Lee, H.K.; Lim, K.H.; Chow, P.K.H.; Kuznetsov, V.A. Tumor‐adjacent tissue co‐expression profile analysis reveals pro‐oncogenic ribosomal gene signature for prognosis of resectable hepatocellular carcinoma. *Molecular oncology* **2018**, *12*, 89-113.

24. Wurmbach, E.; Chen, Y.b.; Khitrov, G.; Zhang, W.; Roayaie, S.; Schwartz, M.; Fiel, I.; Thung, S.; Mazzaferro, V.; Bruix, J. Genome‐wide molecular profiles of hcv‐induced dysplasia and hepatocellular carcinoma. *Hepatology* **2007**, *45*, 938-947.

25. Mas, V.R.; Maluf, D.G.; Archer, K.J.; Yanek, K.; Kong, X.; Kulik, L.; Freise, C.E.; Olthoff, K.M.; Ghobrial, R.M.; McIver, P. Genes involved in viral carcinogenesis and tumor initiation in hepatitis c virus-induced hepatocellular carcinoma. *Molecular medicine* **2009**, *15*, 85.
